# Mutations within lncRNAs are effectively selected against in fruitfly but not in human

**DOI:** 10.1186/gb-2013-14-5-r49

**Published:** 2013-05-27

**Authors:** Wilfried Haerty, Chris P Ponting

**Affiliations:** 1 MRC Functional Genomics Unit, Department of Physiology, Anatomy and Genetics, University of Oxford, Oxford OX1 3PT, UK

## Abstract

**Background:**

Previous studies in *Drosophila *and mammals have revealed levels of long non-coding RNAs (lncRNAs) sequence conservation that are intermediate between neutrally evolving and protein-coding sequence. These analyses compared conservation between species that diverged up to 75 million years ago. However, analysis of sequence polymorphisms within a species' population can provide an understanding of essentially contemporaneous selective constraints that are acting on lncRNAs and can quantify the deleterious effect of mutations occurring within these loci.

**Results:**

We took advantage of polymorphisms derived from the genome sequences of 163 *Drosophila melanogaster *strains and 174 human individuals to calculate the distribution of fitness effects of single nucleotide polymorphisms occurring within intergenic lncRNAs and compared this to distributions for SNPs present within putatively neutral or protein-coding sequences. Our observations show that in *D.melanogaster *there is a significant excess of rare frequency variants within intergenic lncRNAs relative to neutrally evolving sequences, whereas selection on human intergenic lncRNAs appears to be effectively neutral. Approximately 30% of mutations within these fruitfly lncRNAs are estimated as being weakly deleterious.

**Conclusions:**

These contrasting results can be attributed to the large difference in effective population sizes between the two species. Our results suggest that while the sequences of lncRNAs will be well conserved across insect species, such loci in mammals will accumulate greater proportions of deleterious changes through genetic drift.

## Background

Although protein coding sequence occupies a little over 1% of the human genome, approximately 10-fold more non-coding sequence is predicted to have been under purifying selection [1]. For smaller genomes, larger proportions (for example, 50% of all *Drosophila *sequence) have been predicted to have been under selective constraints [13]. These estimates are founded on the assumption that sequence conservation is caused not by low rates of mutation, but instead by the high rates at which deleterious alleles are purged from the population by natural selection, an assumption that is well supported [47].

A considerable fraction of conserved non-coding sequences in human and fruitfly genomes are transcribed [8,9]. Non-coding transcripts can be classified into small RNAs (<200 nt, such as microRNA) and long RNAs (>200 nt, lncRNA). Many lncRNAs are spliced and/or polyadenylated [10], and they show tendencies to contain a smaller number of exons than protein coding genes and to be expressed in a tissue and/or developmental stage-specific manner [11]-[13].

A handful of lncRNAs have been functionally characterised as being involved in dosage compensation in either human (*Xist *[14]) or *Drosophila *(*roX1*, *roX2 *[15]), or having roles in imprinting or chromatin modification (*AIRN *[16]; *HOTAIR *[17]), in alternative splicing regulation or in cell differentiation (*MALAT1*, *Tug1 *[18]-[20]). More broadly many lncRNAs appear to be involved in gene expression regulation in either *cis *or *trans*, through the local modification of chromatin and/or direct interaction with protein complexes, DNA or RNA sequences [11,12,21]-[23]. Recently lncRNAs have also been associated with the maintenance of embryonic stem cell pluripotency [24,25]. Furthermore, there is limited evidence to link some lncRNAs, such as *ANRIL *or *HOTAIR*, to human pathologies [26,27]. However, the functional contribution to biology from the vast majority of long non-coding RNAs (lncRNAs) remains unknown.

If a lncRNA has retained functionality over a long evolutionary time-period then mutations that abolish or diminish the function would be deleterious and would preferentially be purged from the species lineage. This would be reflected in a greater level of sequence conservation between species. Indeed, lncRNAs have been found to be significantly better conserved between species than are putatively neutrally evolving sequences, such as ancestral repeats in mammals [28]-[30] or small introns in *Drosophila *[13]. Furthermore, mammalian lncRNAs are enriched in conserved sequences identified either by elevated conservation (for example, phastCons [2]) scores or by applying a neutral model based on sequence insertions and deletions [28,30]. Additionally, increased conservation of the dinucleotide splice sites and a suppressed transversion rate have also been reported for mammals [28]. However, in each organism analysed thus far, lncRNA sequences have been shown to diverge far more rapidly than have protein-coding sequences [13,28]-[31]. These observations indicate an intermediate state in selective constraints between protein-coding sequences and neutrally evolving sequences. The rapid divergence of lncRNA sequences between species complicates the identification of orthologous sequences for many of the lncRNA loci. Therefore, instead of nucleotide conservation, the conservation of orientation and position relative to an orthologous protein coding-gene can be used to define positionally equivalent lncRNAs between species [13,32].

To date, most evolutionary analyses on lncRNAs have been conducted at the interspecies level using species that diverged approximately 75 million (human - mouse [28]) or 5 million years (*Drosophila melanogaster *- *D. simulans *[13]) ago. Although there is mounting evidence for purifying selection acting on lncRNAs, we note that previous analyses have used only a single reference genome per species. Previous studies reported an increased conservation level relative to a neutral reference [13,28]-[30], but they have not directly determined the strength of selection acting on these non-coding sequences nor do they provide an understanding of the fitness effects of mutations, in terms of the product of the effective population size (*Ne*) and selection coefficient (*s*), occurring within these transcripts.

It is important to compare interspecific indicators of constraint to intraspecific estimates of fitness effects since recent findings have demonstrated rapid evolution of lncRNAs that are specific to individual lineages [33]. A comparison between species can inform on past events but rarely does it have the power to identify contemporaneous or lineage-specific selective constraints. Even when employing comparisons among multiple species it is challenging to ascertain, within a specific lineage, the nature and the strength of the selective pressures acting on rapidly evolving loci.

For instance, the *HOTAIR *locus has evolved rapidly since the last common ancestor of mouse and human and differences in the consequences of knockout in these species' cell lines have been interpreted as indicating the evolution of lineage specific biological functions [34]. Additionally, it was recently demonstrated that expression of a large number of lncRNA loci has altered rapidly among murid lineages [33]. Consequently, a low level of sequence conservation between two species could reflect, at one extreme, a historically low level of sequence constraint in both lineages, or, at the other extreme, it could reflect sequence that is constrained in only a portion of a single species lineage. Deciding among this range of possibilities relies on determining constraint within extant populations, for example by identifying whether derived low frequency alleles are enriched, relative to neutral sequence, within human or *Drosophila *lncRNA sequence [35]. A recent study indicated that this was, indeed, the case for human lncRNAs identified by the ENCODE consortium [36].

In such studies we need to consider that most human variants are recent [7,37], and there is a negative correlation between the age of the variant and its deleterious effect [7]. Consequently the bulk of deleterious mutations within a species are less likely to be detected when comparing distantly-related species as they will not often reach fixation.Therefore inter-species comparison will focus on substitutions events that are at most weakly deleterious as deleterious mutations are rarely fixed. Once again this underscores the importance of analysing, at the population level, nucleotide variation occurring within lncRNA loci if we are to better understand the relationships linking their evolution and function. A potentially important confounding issue that needs to be considered in such analyses is that of background selection as well as selective sweeps, where selection at one site reduces genetic diversity, but not divergence, at linked sites [38]. To account for this effect, variation at tested sites needs to be compared against variation in physically linked putatively neutral sites.

For this study, we have taken advantage of recent high-throughput sequencing projects win *D. melanogaster *[39] and humans [37][40], and the annotation of intergenic lncRNAs in both species [13,41]. The availability of these large population datasets permits polymorphism and divergence distributions to be investigated in both species across both coding and non-coding gene models. If the function of a lncRNA locus is mediated through the act of transcription rather than through the RNA transcript itself [42,43] then we expect no difference in nucleotide conservation between exons and introns. In contrast, if the spliced transcript primarily has a RNA sequence-dependent function then its exonic sequence is expected to be well-conserved relative to its introns, as has been observed for protein-coding genes [44].

Our results reveal hitherto unappreciated distinctions in constraint between lncRNA exons and introns which are abundantly evident for *Drosophila *but are far less so for humans. In *Drosophila *striking differences in conservation between exons and introns suggest that the spliced transcript is often important in mediating the biological functions of lncRNA loci. Our analysis of site frequency spectra indicates that purifying selection has been effective on *D. melanogaster *lncRNA sequence but, importantly, not on human lncRNAs. Selection on mutations within human lncRNAs appear to be effectively neutral as a consequence of our species' unusually low effective population size.

## Results

### Conservation of intergenic lncRNA exons in *Drosophila*

Our previous evolutionary rate analyses of *Drosophila *[13] or mammalian [28,30,45] intergenic lncRNAs considered the degree of constraint associated with transcribed lncRNA sequence under the assumption that small introns and preserved transposable element sequences ('ancestral repeats') evolve neutrally [3,46-48].

**Figure 1:**
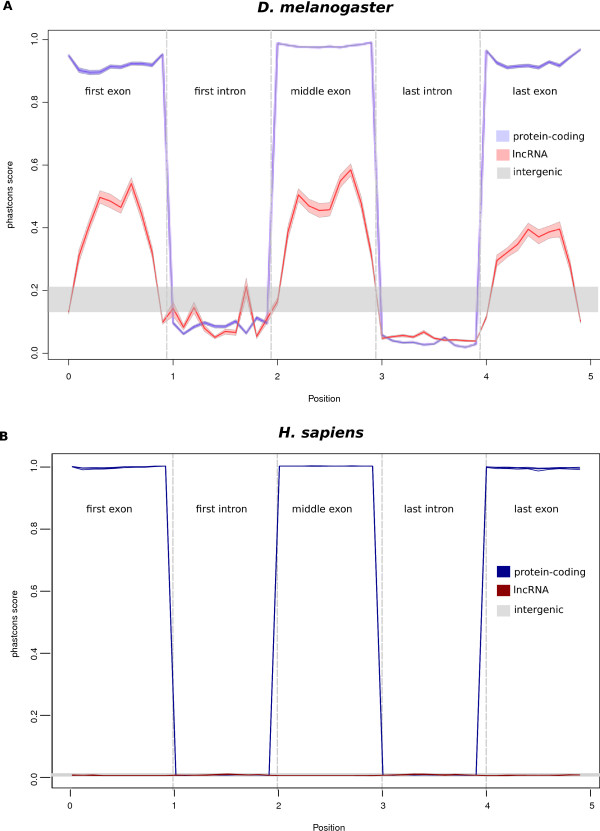
**Median sequence conservation (phastCons) score across protein coding (blue) and lncRNA (red) exons and introns in *D ***. *melanogaster *(A) and in human (B). Non-overlapping windows each comprising 10% of the sequences were used. The shaded areas represent the 95% confidence intervals over the median. The grey lines represent the median scores computed using 1,000 resampling of intergenic sequences matching the lncRNA size distribution.

We extended these analyses firstly by addressing the issue of whether, as for protein-coding sequence [44], exonic sequence is better conserved than intronic sequence. To do this we performed a metagene analysis by recording the median phastCons scores of decile portions for the first, middle or last exons, or their intervening introns, of 1,115 fruitfly and 4,662 human lncRNAs (Figure 1).

For *Drosophila *lncRNAs, we observed a strong contrast in median phastCons scores between their exons and their introns (Figure 1). While protein-coding exons exhibit the greatest degree of conservation, as expected lncRNA exons are associated with intermediate conservation levels, greater than those for protein-coding or lncRNA introns or indeed randomly sampled intergenic sequence (*P*<0.001, Figure 1A). Strong purifying selection in exonic, but not intronic, sequence implies that the molecular functions of these multi-exonic fruitfly lncRNAs are predominantly RNA-sequence specific rather than requiring only the process of transcription, for example during chromatin remodelling [11,42,43].

Performing the identical analysis on a set of human lncRNAs [41] revealed their median phastCons scores to be low not just for introns but also for exons (Figure 1B). There is a significantly greater conservation for lncRNA exons compared with introns (*P *< 0.05) except for the 3′ last-most exon whose conservation is not significantly different to that of introns (*P *>0.05 in all comparisons, Additional File 1). Moreover, sequence conservation in human lncRNA exons or introns is little different from conservation of intergenic sequence. We found similar results when using different human lncRNA sets as well as a set of positionally equivalent lncRNAs between human and mouse (Additional File 2).

Interestingly, when, instead of median values, mean phastCons scores for human lncRNA exons are considered, these are marginally higher than intronic scores (Additional File 1). We conclude from these observations that there is substantial heterogeneity in conservation among human lncRNA loci, yet sequence for the majority of such loci shows little or no conservation.

We noted that *D. melanogaster *lncRNAs exhibit no elevation of phastCons scores at their 5′ or 3′ splice sites using either the median or mean conservation scores (Figure 1A, Additional File 1). To investigate this further we compared the conservation of splice site dinucleotides ('GT' and 'A'G') across five species with randomly selected 'GT' and 'AG' dinucleotides yet found no significant difference in their levels of conservation (Additional File 3). One conceivable explanation is that across the approximate 300 million years of evolution represented in the Diptera and Coleoptera phastCons scores, splice site dinucleotides have been conserved less than over the approximate 450 million years represented in the vertebrate phastCons scores.

### Lowered polymorphism levels within intergenic lncRNA exons relative to introns

The conservation analysis that we present above illustrates qualitatively the relative conservation between exons or introns, and differences in constraint between fruitfly and mammalian lncRNA sequences. This analysis is based on aligned sequences from highly divergent species and therefore provides us with evidence on past selection but unfortunately not on more contemporary evolutionary processes. To address this, we looked to DNA polymorphism data from both *D. melanogaster *and human populations.

We considered 2,263,316 polymorphic sites in *D. melanogaster *and 12,640,342 in human, and used pairwise alignments with *D. simulans *and *D. yakuba*, or with *P. troglodytes *and *M. mulatta*, respectively to polarise SNPs for *D. melanogaster *or human according to whether they were ancestral or derived using maximum parsimony (Table 1). For all subsequent analyses, we compared observed levels of polymorphism and divergence within lncRNA loci to polymorphism and divergence observed within putatively neutrally evolving sequences such as small introns (< 86*nt*) in *Drosophila *[3,46,48] and ancestral repeats in human [47]. Importantly, in order to take into account potential variation in local rates of mutation and/or substitution as well as nucleotide content in human or *Drosophila*, we limited our analyses to just those protein-coding genes that flank intergenic lncRNAs. Additionally, we considered only small introns present within protein coding genes that are direct neighbours and within 5 kb of lncRNA loci in *D. melanogaster *and only ancestral repeats found within intergenic sequences that are direct neighbours of mammalian lncRNA loci. We retained only those lncRNA loci for which matching small introns or ancestral repeats could be identified.

**Table 1:** Number of polarised polymorphic sites among 162 *D*.

Feature	*D. melanogaster*	*H. sapiens*
Total	2,263,316	12,640,342
lncRNA exons	29,535	49,505
Ancestral repeats	-	317,098

Others	921,066	8,039,366

*melanogaster *strains and among 174 humans of African origin. The ancestral and derived states for each SNP were defined using alignments of *D. melanogaster *with *D. simulans *and *D. yakuba *and of *H. sapiens *with *P. troglodytes *and *M. mulatta*.

For both human and *Drosophila*, we observed a lower density of polymorphic sites within protein-coding exons than in introns (*P *<0.001 in both species), which indicates strong negative selection having acted on these exons. Although similar trends were observed for lncRNAs, differences in SNP densities for lncRNA exons and introns were not significant (P >0.05 in both species, Tables 2 and 3).

**Table 2:** Average (standard deviation) polymorphism estimates for 1ncRNA loci and their flanking protein coding genes (within 5 kb) in *D *.

Upstream coding	4.8 × 10^-3 ^(4.4 × 10^-3^)	5.39 × 10^-3 ^(3.8 × 10^-3^)	-0.36 (0.97)	0.095 (0.74)
lncRNA exons	4.94 × 10^-3 ^(3.2 × 10^-3^)	5.88 × 10^-3 ^(3.2 × 10^-3^)	-0.53 (0.81)	0.064 (0.072)
Small introns	1.01 × 10^-2 ^(1.12 × 10^-2^)	8.96 × 10^-3 ^(8.16 × 10^-3^)	0.15 (1.17)	0.115 (0.10)

*melanogaster*.

**Table 3:** Average (standard deviation) polymorphism estimates for 1ncRNA and their flanking protein coding genes in human.

Upstream coding	1.05 × 10^-3 ^(1 × 10^-3^)	1.03 × 10^-3 ^(0.07 × 10^-4^)	0.003 (0.91)	1.51 ×^-3 ^(1.74 × 10^-3^)
lncRNA exons	1.06 × 10^-3 ^(8.85 × 10^-4^)	1.16 × 10^-3 ^(6.91 × 10^-4^)	-0.21 (0.99)	1.59 ×^-3 ^(1.58 × 10^-3^)
Upstream lncRNA	1.09 × 10^-3 ^(1.07 × 10^-3^)	1.19 × 10^-3 ^(8.26 × 10^-4^)	-0.14 (0.92)	1.64 ×^-3 ^(1.79 × 10^-3^)
PE lncRNA exons	9.73 × 10^-4 ^(7.87 × 10^-4^)	1.13 × 10^-3 ^(6.61 × 10^-4^)	-0.27 (0.88)	1.46 ×^-3 ^(1.41 × 10^-3^)
PE lncRNA introns	1.04 × 10^-3 ^(7.62 × 10^-4^)	1.08 × 10^-3 ^(4.67 × 10^-4^)	-0.20 (0.77)	1.42 ×^-3 ^(9.2 × 10^-4^)
Controls lncRNA exons	1.04 × 10^-3 ^(8.62 × 10^-4^)	1.15 × 10^-3 ^(6.57 × 10^-4^)	-0.22 (0.85)	1.46 ×^-3 ^(1.54 × 10^-3^)
Controls lncRNA introns	9.84 × 10^-4 ^(6.48 × 10^-4^)	1.08 × 10^-3 ^(5.09 × 10^-4^)	-0.26 (0.75)	1.47 ×^-3 ^(1.33 × 10^-3^)
Ancestral repeats	1.51 × 10^-3 ^(1.81 × 10^-3^)	1.68 × 10^-3 ^(1.14 × 10^-3^)	-0.13 (0.92)	2.34 ×^-3 ^(3.48 × 10^-3^)

PE: position equivalent.

**Figure 2:**
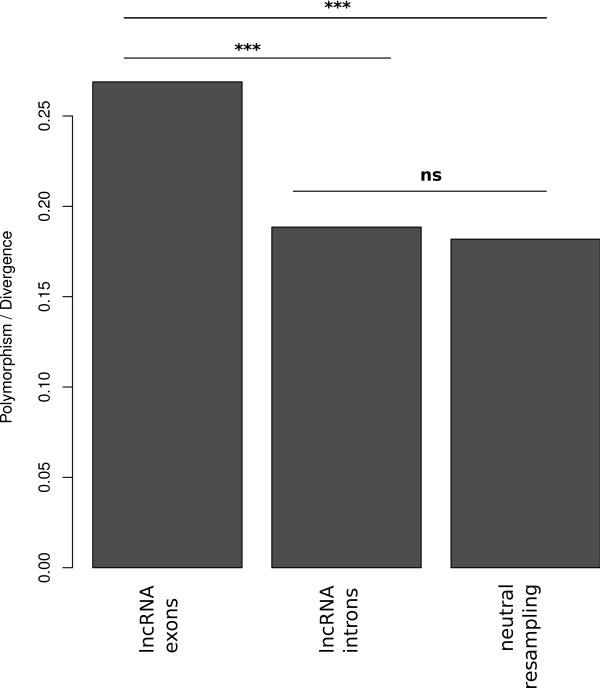
**McDonald-Kreitman test for lncRNA exons and introns**. Small intronic sequences (≤86 nt, disregarding the first 6 nt and last 16 nt) were used as a proxy for neutrally evolving sequences. *** *P *<0.001, ns: not significant.

The ratio of *D. melanogaster *polymorphism to *D. melanogaster*-*D. simulans *divergence within lncRNA exons or introns was compared to that of small introns or randomly sampled flanking intergenic sites. The significant excess of polymorphism with respect to divergence within lncRNA exons (*χ*2 test, *P *<0.001), but not introns (*χ*2 test, *P *>0.05, Figure 2), illustrates the strength of purifying selection acting on fruitfly lncRNAs, and specifically their exons.

### Evidence for strong purifying selection on intergenic lncRNAs in *Drosophila*

Next, to test for the strength of selection within exons or introns from fruitfly or human lncRNA loci, we compared the nucleotide variation within lncRNAs and protein coding exons and introns to putatively neutral sequences using the average number of pairwise nucleotide differences per sites (*πT *, *θW *[49,50]), and Tajima's D [51] which tests for departures from neutrality. We also assessed the nucleotide divergence between *D. melanogaster*-*D. simulans*, and human-macaque using the Jukes-Cantor corrected divergence (*k *[52]).

As expected, we inferred stronger selective constraints on the protein-coding exons and introns of fruitfly genes, owing to their lower Tajima's D and divergence (*k*), than for small introns, our neutral evolution proxy (Kruskal-Wallis test, *P *<0.05 in all comparisons, Table [2]). Likewise, *D. melanogaster *lncRNA exons and introns were associated with lower Tajima's D and *k *values relative to our neutral sequence proxy, namely small introns (*P *<0.001 in both comparisons). Greater selective constraint on *Drosophila *lncRNA exonic sequence was observed: values for lncRNA exons were significantly lower than for lncRNA introns (*P *≤0.01 in both comparisons). Although we found no difference in *πT *, *θW *or Tajima's D values between lncRNAs and protein coding upstream sequences (*P *>0.05 in all comparisons), we found lncRNA upstream sequences to be less diverged than those of protein coding sequences (*P *<0.001). This observation of lower interspecific divergence is likely to be the consequence of lncRNA gene models being incomplete, which in turn is a consequence of their low expression levels.

Like fruitflies, human protein coding exons are under stronger selective constraints than either lncRNA exons, introns or protein-coding introns as indicated by lower *πT *, Tajima's D and *k *values (*P *<0.001, Table [3]). In contrast to *Drosophila*, we found no significant difference in Tajima's D values computed for human lncRNA exons, introns and their flanking ancestral repeats. Additionally intergenic lncRNAs that are positional equivalents between human and mouse do not show a significant reduction of polymorphism or Tajima's D value relative to a control set of intergenic lncRNAs (*P *>0.05, Table [3], Additional Files 5 and 6).

#### Excess of low frequency variants in Drosophila intergenic lncRNAs relative to neutral sequences

**Figure 3:**
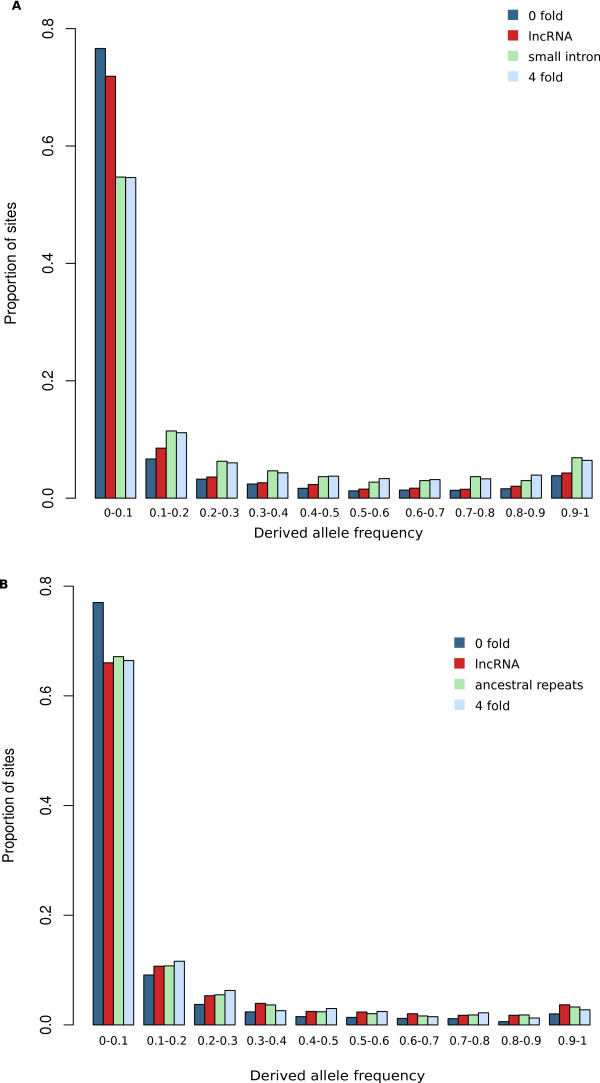
**Comparison of derived allele frequency distribution of SNPs at 0-fold degenerate sites (blue), lncRNA exons (red), neutrally evolving sequences: small introns - ancestral repeats (green) and four-fold degenerate sites (light blue), for *D ***. *melanogaster *(A) and human (B).

We next compared the derived allele frequency spectra of polymorphic sites within fruitfly lncRNA exons to those within small introns. This revealed that lncRNA exons have a significantly higher proportion of SNPs with low frequency (≤0.01) derived alleles (Kolmogorov-Smirnov test, *P *<0.001). This indicates that they have been subject to a greater degree of purifying selection in these fruitflies' recent evolution, since their divergence with *D. simulans *(Figure 3). This effect was not solely due to a G+C enrichment of conserved non-coding regions relative to non-conserved non-coding regions [53] since significant enrichment for low frequency derived alleles was observed for both G:C →A:T and A:T→G:C substitutions in lncRNA exons (Kolmogorov-Smirnov tests *P *<0.001 in both comparisons) relative to small introns. The strength of purifying selection for fruitfly lncRNA exons appears to be lower than for non-synonymous or 3′ UTR SNPs in protein-coding transcripts but stronger than for SNPs in their 5′ UTRs or four-fold degenerate sites (Additional File 7). We observed that sequences upstream of the lncRNA loci in *D. melanogaster *are also enriched in low frequency variants relative to small introns or to upstream sequences of protein-coding genes (Additional File 8). This could reflect purifying selection acting on these elements and/or the presence of unannotated upstream lncRNA exons.

An equivalent analysis on the set of human lncRNAs, using data from the 1000 Genomes Project [40], revealed no enrichment of rare variants within human lncRNA exons relative to candidate neutrally evolving sequences such as four fold degenerate sites, introns or ancestral repeats (*P *>0.05, Figure 3). This result is important in allowing us to extend from our previous observation of a low degree of conservation between species, to effectively neutral or weak negative selection occurring since the emergence of modern humans. We similarly found that the derived allele frequency (DAF) of SNPs within positionally conserved lncRNAs does not depart significantly from the distribution observed for neighbouring ancestral repeats. While we observe a departure in the human lncRNA SNP DAF with respect to that for ancestral repeats sampled genome-wide, this is likely attributable to the effects of background selection: negative selection acting on the genomically proximal protein-coding genes.

#### Deleterious effect of mutations within intergenic lncRNAs in fruitfly but not in human

In our final analysis we estimated the distribution of fitness effects of new mutations within *D. melanogaster *or human lncRNA exons from their respective site frequency spectra. Because the DAF spectra can be influenced by past variation in effective population size, we employed the method of Keightley and Eyre-Walker [54] that estimates the distribution of fitness effect of new mutations and demographic parameters from the folded frequency spectrum.

**Figure 4:**
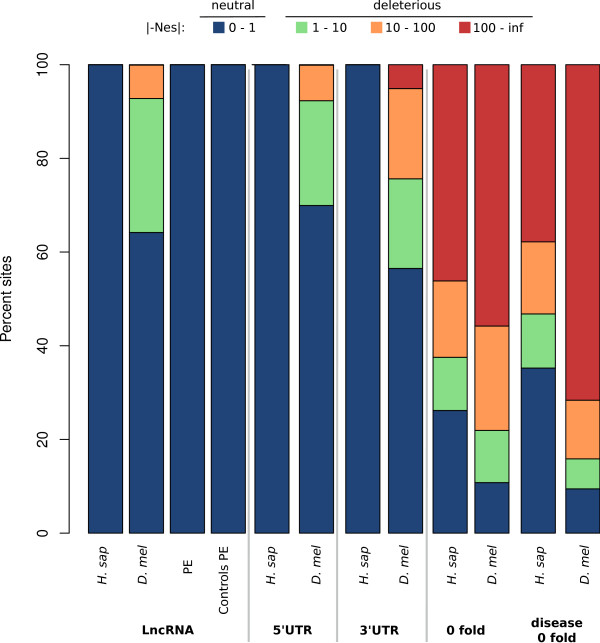
**Comparison of the distribution of fitness effect of mutations occurring within lncRNAs in D**. melanogaster and human. For comparison the distributions were also computed for the 5′ and 3′ UTRs of protein-coding genes in *D. melanogaster *and human, as well as for 0-fold degenerate sites at genes annotated in the OMIM database or with lethal mutant phetnotype in *D. melanogaster*, and for 0-fold degenerate sites from the remaining non-annotated protein-coding genes. The distributions were computed using DFE alpha [101], the values represent the average over 200 boostraps for each locus in each category.

As our proxy for neutrally evolving sequence we considered site frequency spectra from sites randomly sampled within flanking intergenic sequences. Likewise, we used four-fold degenerate sites as neutral proxy when calculating the distribution of fitness effect of new mutations at 0-fold degenerate sites. In fruitflies, two-thirds of mutations in lncRNA exons are predicted to be effectively neutral (*Nes *<1; 64.18%, 95% CI 63.8% to 64.5%) while one-third are likely to be deleterious (*Nes *>1; 35.82%, 95% CI 35.0% to 36.6%). In stark contrast, no mutations in human lncRNAs were classified in this analysis as being deleterious, including those lncRNAs with positional equivalents in mouse. Consequently, we predict that the great majority of substitutions in human lncRNA sequence are effectively selectively neutral or nearly neutral (Figure 4). As an additional comparison we also computed the distribution of fitness effect for non-degenerate sites within protein-coding genes associated with lethal mutant phenotypes in *D. melanogaster *or associated with genetic diseases or syndromes in human. As expected for these two sets of sites we observed an increased proportion of sites classified as being highly deleterious (*Nes *>100) relative to non-degenerate sites from all remaining protein-coding genes. Once again the proportion is strikingly higher for the *D. melanogaster *set (70.92%) than it is for the human set (59.74%) of deleterious amino acid changes.

Our estimates of the distribution of fitness effects of newly arising mutations within non-degenerate sites are in agreement with previous analyses conducted in human. Boyko *et al. *[55] as well as Keightley and Eyre-Walker [54] identified between 22% and 34% of newly arising mutations within the African population as being selectively effectively neutral (our estimate: 26.69%).

## Discussion

Previous between species comparisons predict lncRNAs to have evolved under a regime of purifying selection that is considerably weaker than for protein-coding sequences [13,28]-[31]. Because of their design, virtually all of these experiments consider evolutionarily ancient selective events. However by taking advantage of available sequenced genomes of individuals from within the same species, we can now: (1) infer the evolution of these sequences at a considerably shorter time scale; (2) quantify more precisely the strength of recent or contemporaneous selection acting on lncRNAs; and (3) assess the distribution of fitness effect of new deleterious mutations occurring within these sequences. From the reported importance of a limited subset of lncRNAs in gene regulation [23,25,26], it might have been expected that human lncRNAs would exhibit a weak signature of purifying selection at the population level.

### *D. melanogaster *intergenic lncRNA evolution

Our results show that *D. melanogaster *intergenic lncRNAs are subject to moderately strong selective constraints. SNPs occurring within fruitfly lncRNAs are characterised by an excess of rare variants relative to neutral sequences (either small introns or randomly sampled sites within flanking intergenic sequences), leading to a negative estimate of Tajima's D, and a L-shaped site frequency spectrum. We reached the same conclusion when considering the minor allele frequency or the derived allele frequency or when taking account of mutational biases (AT→GC, GC→AT). Although this effect could be explained by a recent population expansion [56], we reached identical conclusions when using an algorithm that estimates population parameters before testing for the distribution of fitness effect of newly arising mutations [54,57].

Our findings of fruitfly lncRNA constraint at the population level are confirmed at the interspecific level by comparing nucleotide conservation between lncRNA exons and introns, an extension to our previous findings [13]. LncRNA exons were shown to exhibit an intermediate level of conservation between protein-coding exons and intergenic sequences, while conservation of lncRNA introns does not differ significantly from that of intergenic sequence.

These differences in conservation between *Drosophila *lncRNA exons and introns, as well as the observation of a greater proportion of low frequency variants within lncRNA exons relative to lncRNA introns, argue strongly for spliced transcripts being important for the function of many fruitfly lncRNAs and not RNA sequence-independent biological function as found for some lncRNA loci such as *HSI *and *Airn *[42,43].

In contrast to results for human lncRNAs (which confirm our previous observations [28,31]) we found no significantly increased conservation for splice sites in *Drosophila *lncRNAs relative to randomly selected 'GT' and 'AG' dinucleotides within intergenic and intronic sequences. This lack of increased splice site conservation, despite an increased nucleotide conservation of the lncRNA exons, may indicate a rapid divergence of splicing elements within these long non-coding RNAs. This observation could, however, also result from the mis-annotation of splice sites as a consequence of typically low sequence coverage for lncRNA models in RNA-Seq experiments.

### Human intergenic lncRNA evolution

In contrast to evidence in flies, we found no evidence from human population data for widespread purifying selection acting on lncRNA sequence, and only a weak signal of elevated sequence conservation between vertebrate species. Few human lncRNAs were as highly conserved as those from *Drosophila *(Additional File 9).

As evidence for lncRNA sequence conservation across species is scarce, potentially orthologous transcripts transcribed with the same orientation and syntenic position relative to an orthologous protein coding locus have been identified among human, mouse and zebrafish [32]. If such positionally equivalent lncRNAs are orthologous and retain ancestral function then purifying selection acting on these loci might be expected to be stronger than for the remaining lncRNAs. However, these positionally equivalent lncRNAs' sequence conservation across vertebrates, as well as their site frequency spectra, were found not to differ from those of a control set of human lncRNAs. Once again this highlights the weak selective constraints that have acted both recently and more historically on vertebrate lncRNAs. Accordingly, zebrafish lncRNAs with positional equivalents in human or mouse were found not to exhibit sequence conservation between these species [32].

The lack of evidence for strong or widespread purifying selection or the weak selective effect of mutations within non-coding sequences in human has been reported previously, although not specifically for transcribed non-coding sequence. Torgerson *et al. *[58] compared polymorphisms in human within conserved intergenic sequences (>5 kb upstream and downstream of annotated transcripts) with synonymous site polymorphisms and found no evidence for selection on intergenic conserved sequences. Likewise, Krukyov *et al. *[59] and Chen *et al. *[60] found that despite purifying selection acting on the most conserved non-coding elements in human, of mutations within them have only weak effects on fitness.

### Why might fly intergenic lncRNA evolution differ from human intergenic lncRNA evolution?

We estimated that an average of 35.82% of new mutations within *D. melanogaster *intergenic lncRNAs are effectively negatively selected. However, selection on all mutations within human intergenic lncRNAs, even those with a positional equivalent in mouse, was predicted to be effectively neutral.

Some of the observed differences in conservation and selection acting on lncRNAs between *D. melanogaster *and humans could be due to different origins of the two datasets. Our set of human lncRNAs was derived from adult tissues [41] whereas the fruitfly lncRNAs were identified from a developmental time course gene-expression analysis [9,13] and could therefore be subject to stronger selective constraints. Previous studies showed increased purifying selection on protein-coding genes expressed early during development relative to genes expressed during the adult stage [61].

A second explanation for the observed differences between *D. melanogaster *and human lncRNAs in conservation and allele frequency distribution relates to differences in the effective population sizes of the two species. The influence of effective population size on the probability of fixation of a deleterious mutation is well documented [62]. According to the nearly neutral theory of molecular evolution,the probability of fixation of such a mutation is a function of 4*Neμs *(*μ*: mutation rate, *s*: selection coefficient), and thus a weakly deleterious mutation will be effectively neutral if the product of its selection coefficient (*s*) and the effective population size (*Ne*) is near to one [63-65]. There is a considerable difference in estimated effective population sizes of *D. melanogaster *or *H. sapiens*: 1,450,000 *versus *1,200-15,000, respectively [66-68]. This results in a wide range of low selection coefficients *s *for which deleterious mutations have widely varying fixation probabilities between the two species. A deleterious mutation with a small selection coefficient in human is likely to evolve essentially neutrally, while a mutation with the same selection coefficient in *Drosophila *will tend to be subject to stronger purifying selection. More formally any mutation with |*s*| > 1/*Ne human *will be under the scrutiny of selection in either species while any mutation with 1/*Ne human *> |*s*| > 1/*Ne Drosophila *will be under a selectively near neutral regime in human but will be under more effective negative selection in *D. melanogaster*. According to the effective population size estimates cited above, the minimum value of *s *for selection to act on deleterious variants ranges from approximately 7 × 10-5 in human to three orders of magnitude lower, 7 × 10-8 in *D. melanogaster*. This difference in effective population size between human and *Drosophila *is a likely explanation of the striking differences in the DAF distributions of variants within lncRNAs in *D. melanogaster *and human.

A third explanation might be that the repertoires of fruitfly or human lncRNA molecular mechanisms are very different, leading to differences in the signatures of selection in their lncRNA sequences. If this is indeed the case then we speculate that fruitfly lncRNA mechanisms will be more critical to its biology than are lncRNA mechanisms to human biology.

From these results testable predictions can be made regarding the evolution and conservation of lncRNA sequences. Deleterious mutations with a particular value of *s *within lncRNA in species with large effective population size, such as insects [59,69], are more likely to be purged leading to a greater sequence conservation. In contrast within species with low effective population size, such as human, weakly to mildly deleterious mutations are more likely to be fixed leading to a greater turn-over of non-coding transcribed sequences [33]. This effect explains the difference in the distribution of fitness effects of deleterious mutations at genes annotated with disease/lethal phenotypes in human and fruitflies.

### Comparison with Ward and Kellis [36]

Our conclusion that negative selection is highly inefficient within human lncRNA variants appears to be at odds with evidence from Ward and Kellis that their variants exhibit a lower mean DAF than genomic samples [36]. This apparent discrepancy could not be explained by the different lncRNA sets being considered. This was because results from our reanalysis of the Ward and Kellis lncRNA set from ENCODE were equivalent to those we report above. It could also not be explained by Ward and Kellis' [36] consideration only of SNPs of Yoruba origin, since when we re-ran our approach using only Yoruba SNPs, no substantive differences were found (Additional Files 10 and 11). Instead, we believe the discrepancy likely arises from the differences in the choice of proxy for neutral sequence. In our analysis, we account for the otherwise potentially confounding factors of background selection and mutational variation by considering sites either within ancestral repeats that flank lncRNA loci, or within flanking intergenic sequence that has been masked for conserved sequence. By contrast, the approach of Ward and Kellis [36] samples sites from concatenated unannotated intergenic sequences drawn from across all autosomes, and thus does not account for background selection or mutational rate variation.

Although interspecies sequence conservation over long evolutionary time is rightly considered as an indicator of functionality, the lack of conservation within lncRNAs does not necessarily imply their lack of functionality [70]. Sequences encoding heart enhancers have been found to be as poorly conserved as randomly sampled sequence [71]. The accumulation of weakly to mildly deleterious mutations within poorly conserved sequence, such as human lncRNA loci, raises the question of how a population can carry an ever increasing burden of deleterious variants within loci that regulate gene expression? Previous hypotheses proposed that such sequences interact with only a limited number of factors or that only a very restricted proportion of sequence is required to convey biological function [70]. Others suggest that compensatory mutations within the locus maintain secondary structure [72] or similarly within the sequence of its interacting partner maintain molecular function. Such compensatory mechanisms [34] and network redundancy have been proposed to explain the rapid sequence evolution of lncRNAs and the absence of mutant phenotypes for some lncRNA knockout models. Finally, the accumulation of slightly deleterious mutations could also be explained by synergistic epistasis, when interactions between mutations produce a greater effect than expected from the sum of their independent effects. This hypothesis was first proposed to explain the mutational load paradox in species with low effective population sizes [73] but may also help to explain the accumulation of potentially deleterious mutations at synonymous sites [74] and within conserved non-coding sequences [59].

The inefficiency, or low degree, of selection acting on mutations within human lncRNAs suggests that for the great majority of these loci extensive phenotyping will be necessary to identify the potential deleterious effects of their disruption. Accordingly, several recent studies have reported that despite phenotypes being observed in cell-based assays for several lncRNA loci (*HOTAIR*, *Malat1*, *Neat1*), no overt phenotype (for example, litter size, body weight or viability) was found in the knockout mice under normal laboratory conditions ([34,75-78]).

However an absence of overt phenotype in laboratory conditions does not necessarily imply that there is no deleterious effect of the knockout. Although the knockout mice did not differ from the wild-type individuals, further analyses found evidence for phenotypes for *Evf2 *[79], and *Bc1 *[80,81] mutants. Analyses in yeast and in worm have revealed that despite the observation of a lack of phenotype for a vast majority of the knockout mutants, fitness effects measured as population growth under a wide range of conditions are apparent for up to 97% of *Saccharomyces cerevisiae *genes [82] and between 42% and 60% of genes assayed in *Caenorhabditis elegans*. Finally, because lncRNAs are most often expressed at low levels in a developmental stage and/or tissue specific manner this increases the difficulty of identifying potential phenotypes associated with their disruption.

## Conclusions

Genetic drift appears to be the main driving force in the evolution of intergenic lncRNAs, at least in humans, as a consequence of our small effective population size. Therefore, weakly to mildly deleterious mutations are likely to have accumulated rapidly within intergenic lncRNAs. The consequences of such an accumulation on lncRNA function and on human biology have yet to be experimentally assessed. Our observations serve to highlight the pressing need for extending the study of these loci to *in-vivo *systems combined with extensive phenotyping. Our results support a less prominent biological role for many of these non-coding loci than has been proposed previously [83,84].

## Materials and Methods

In all analyses that we describe below, calculated *P *values were corrected for multiple testing using a Bonferroni correction [85].

Our analysis in *D. melanogaster *was conducted on the set of 1,115 long non-coding intergenic RNAs defined by Young *et al. *[13] using polyA+-selected transcriptome data from the ModEncode Project [9] having excluded four loci owing to their overlap with recently predicted small open reading frames [86]. For comparison we also analysed a set of 4,662 human lncRNAs identified by Cabili *et al. *[41] from polyA+-selected libraries using conservative criteria, namely one isoform reconstructed in at least two tissues or by two assemblers [41].

Because mono-exonic lncRNAs models are not stranded, we limited our analysis to multi-exonic loci. Furthermore, in order to avoid the confounding effects arising from selection acting on protein-coding genes we focused our analysis on intergenic lncRNA loci, instead of intronic, antisense or lncRNAs that overlap untranslated regions of protein-coding genes.

We used the mouse lncRNAs annotated by Ensembl and by Belgard *et al. *[87] to identify positional equivalent lncRNAs between mouse and human. Using protein-coding genes with 1-to-1 orthologous relationships between human and mouse and flanking a lncRNA locus in both species, we defined as positional equivalents those lncRNAs that were found in the same transcriptional orientation and the same location relative to a protein-coding gene in both species. Furthermore, in order to take into account potential selection acting on the nearby protein-coding gene, we also identified a control set composed of lncRNAs flanking protein-coding genes with 1-to-1 orthologs but with different transcriptional orientations and/or positions relative to the protein coding gene. We identified 374 positional equivalents loci between human and mouse, and 802 control lncRNAs.

We collected 2,993 genes described as being involved in syndromes and genetic diseases from OMIM database [88,89]. Using the FlyBase database [90], we collated 2,125 genes with lethal mutant phenotypes.

*D. melanogaster *and human gene annotations and genomes were downloaded from FlyBase [90] (release 5.39) and Ensembl [91] (release 64), respectively.

Polymorphism data for 162 *D. melanogaster *strains from Raleigh, North Carolina were downloaded from the *Drosophila *Genetic Reference Panel [39,92,93]. Sites covered by at least 10 reads and without base ambiguity in at least 150 strains were retained for further analysis. A total of 3,172,754 sites across the five major chromosomal elements were used for analysis. For the human dataset, we discarded SNPs within 10 bp of indel calls and chose a quality score threshold to give a 0.1% FDR. The allele frequencies for polymorphic sites were retrieved from the 1000 Genomes Project data. We collected 18,745,840 SNPs in 174 individuals of African origin (a highly polymorphic population) called by the 1000 Genomes Project Consortium [40,94].

For both datasets, we polarised the alleles into ancestral or derived states using the pairwise alignments of *D. melanogaster *with *D. simulans *and *D. yakuba*, and of *H. sapiens *with the chimpanzee (*Pan troglodytes*) and macaque (*Macaca mulatta*) which are available from the UCSC genome database website [95]. We used maximum parsimony to infer the ancestral state of each site, and ambiguous sites were removed from the final dataset. Using genome annotations, we collated sites found within exons and introns of protein-coding genes, lncRNA loci or intergenic sequences or ancestral repeats (transposable elements shared between human, mouse and rat) (Table [1]).

### Evolutionary rates and sequence conservation

PhastCons scores [2] computed using the alignments of 11 *Drosophila *species, *Anopheles gambiae*, *Tribolium castaneum *and *Apis mellifera *(whose divergence spans approximately 300 Mya) were downloaded from the UCSC database [95]. We computed the median phastCons scores for for each of 10 successive windows that each represents a 10% portion of lncRNA exon or intron sequence; exons or introns were further subdivided into 'first', 'middle', 'last' or 'unique' classes with respect to their genomic position. We also collected 1,000 nt of 5′ and 3′ flanking intergenic sequences for both lncRNAs and protein coding loci.

We computed, for each window, 95% confidence intervals using 10,000 bootstraps. As a control, we randomly selected intergenic sequences lying away (>1 kb) from any annotated gene whose size distribution matched that of the lncRNA exons or introns. One thousand such sets of control sequences were defined to permit confidence intervals to be calculated. For comparison this analysis was also performed on the set of protein-coding genes that flank lncRNA loci.

This procedure was repeated for human lncRNA loci and their neighbouring protein-coding genes using phastCons scores computed using the alignments of 46 vertebrate genomes from the UCSC database [95] (approximately 400*My*).

In order to assess the difference in nucleotide conservation between lncRNA exons and introns, we implemented a resampling analysis in which we randomly sampled a single site per feature (exon or intron) within a locus. In total, 1,000 resampling analyses were performed.

We estimated the conservation of the splice sites of both protein-coding and lncRNA loci in flies using the sequence alignments of 50 nucleotides upstream and downstream of the *D. melanogaster *splice sites with *D. simulans*, *D. sechellia*, *D. yakuba *and *D. erecta*. For 5′ and 3′ splice sites and the 20 adjacent intronic sites of protein coding genes and lncRNA loci we computed the information content using the Shannon-Weaver index.

As control, we randomly selected 'GT' and 'AG' dinucleotides within intergenic sequences flanking the lncRNA loci and applied the same procedure.

### Polymorphism estimators

We used VariScan [96] to compute polymorphism indicators (*πT *, *θW *, Tajima's D). Genomic alignments with *D. simulans *and rhesus macaque for *D. melanogaster *and human, respectively, were used to compute the Jukes-Cantor corrected per site divergence (*k*). To avoid any potential bias arising from local variations in recombination rate, mutation rate, efficacy of selection or nucleotide composition, we limited our analysis to only those protein coding genes, small introns or ancestral repeats that are found in the neighbouring genomic regions of lncRNA loci (within 5 kb). Likewise in human we analysed lncRNA loci flanked by proximal (≤10 kb) ancestral repeats and their flanking protein-coding genes. Similar conclusions were reached from analyses with distance thresholds of 5 kb and 20 kb (Additional Files 5 and 6).

Similarly we compared the derived allele frequency of polymorphic sites within lncRNA exons or lncRNA introns to sites within small introns, non-degenerate sites and four-fold degenerate sites.

Because the putatively neutral sites we used are not interdigitated with our sites of interest (such as lncRNA exonic nucleotides), there remains the possibility that our indicators of purifying selection are artificially inflated [97]. In order to take such biases into account, when considering N sites from each lncRNA locus associated with an intergenic flanking sequence (≥1,000 nt following the masking of conserved non-coding elements with nucleotide identity ≥90% over ≥20 nt), we randomly sampled this number N sites from this masked flanking sequence to be used as a neutral proxy. For the study of non-degenerate sites, we used four-fold degenerate sites within the same protein as a neutral proxy in human. However, because there is evidence for selection having acted on four-fold degenerate sites in *Drosophila*, we instead used small introns (≤86 nt) as our neutral proxy and limited our analysis to just those protein-coding genes which contain such small introns. This analysis permits the strength of selection acting on lncRNAs to be estimated while controlling for variations in the local mutation rate, as well as background selection associated with nearby functional elements including protein-coding genes and well conserved non-transcribed non-coding regulatory elements. We used this methodology to assess the degree of selective constraints acting on intergenic lncRNAs through a generalised McDonald-Kreitman test [98-100]. We compared the numbers of polymorphic over divergent sites within lncRNA exons and lncRNA introns to the numbers observed within sampled putatively neutral sites using a *χ*2 test with one degree of freedom.

For either *D. melanogaster *or human lncRNAs, we used the site frequency spectra of mutations occurring within the sampled putatively neutral sites to estimate the distribution of fitness effect of new deleterious mutations within lncRNAs (in terms of -*Nes*) using DFE-alpha [54,57,103]. Confidence interval values for the proportion of sites under the different *Nes *categories were estimated through 200 bootstraps per locus. This analysis should therefore also take into account the effects of background selection as for each locus a 'neutral' reference is drawn from the same region.

### Statistics

Comparisons between locus classes for the polymorphism estimators were performed using Kruskal-Wallis tests. The minor and derived allele frequencies distributions for each class were compared using Kolmogorov-Smirnov tests.

## Abbreviations

DAF: derived allele frequency; DFE: deleterious fitness effect; FDR: false discovery rate; lncRNA: long non-coding RNA; SNP: single nucleotide polymorphism.

## Authors' contributions

WH and CPP conceived and designed the study. WH performed the analyses and statistics. WH and CPP wrote the manuscript. Both authors read and approved the final manuscript.

## Supplementary Material

Additional File 1 Average phastCons scores across protein-coding (blue) and lncRNA (red) gene models in *D. melanogaster *(A) and human (B, C). Two hundred evenly-spaced nucleotides were randomly sampled per feature. The gray lines represent the 95% confidence intervals computed over 1,000 resampling. Average phastCons score for lncRNAs in human was computed over 200 randomly selected equidistant nucleotides within each of the categories. Confidence intervals were computed using 1,000 resampling of the data. Click here for file

Additional File 2 **Median sequence conservation (phastCons) score across protein coding (blue) and positionally equivalent (PE) lncRNA (red) in human**. Click here for file

Additional File 3 Comparison of protein-coding (blue) and lncRNA (red) 5' (A) and 3' (B) splice site conservation in *D. melanogaster *. Only protein coding sequences flanking lncRNAs were used in the analysis. The control set is based on the random selection of 'GT' and 'AG' dinucleotides within the intergenic sequence flanking the lncRNAs in *D. melanogaster*. The Shannon-Weaver index was computed for each site using the alignments of each splice site and its neighbouring sequences with *D. simulans*, *D. sechellia*, *D. yakuba *and *D. erecta *with Muscle [102]. Click here for file

Additional File 4 Distribution of the distances between consecutive SNPs within protein coding (black) and lncRNA (red) exons in *D. melanogaster*. Click here for file

Additional File 5 **Average (standard deviation) polymorphism estimates for lncRNA and their flanking protein coding genes in human**. PE: positional equivalent. A maximum distance threshold between lncRNA loci and ancestral sequences of 5 kb was applied. Click here for file

Additional File 6 **Average (standard deviation) polymorphism estimates for lncRNA and their flanking protein coding genes in human**. PE: positional equivalent. A maximum distance threshold between lncRNA loci and ancestral sequences of 20 kb was applied. Click here for file

Additional File 7 Comparison of derived allele frequency distribution of SNPs at non-synonymous sites (dark blue), within 3′ UTR (yellow), lncRNA exons (red), 5′ UTR, at four-fold degenerate sites (light blue), and within small introns in *D. melanogaster*. Click here for file

Additional File 8 Derived allele frequency spectra for 0-fold, four-fold degenerate sites, sites within lncRNA, sites upstream (400 nt) lncRNAs and protein coding genes in *D. melanogaster *(A) and human (B). Click here for file

Additional File 9 **Distribution of average conservation scores for intergenic lncRNAs in human**. Click here for file

Additional File 10 **Comparison of derived allele frequency distribution of SNPs at 0-fold degenerate sites (blue), GENCODE lncRNA exons (red), ancestral repeats (green) and four-fold degenerate sites (light blue) in human**. Click here for file

Additional File 11 **Comparison of derived allele frequency distribution of SNPs at 0-fold degenerate sites (blue), GENCODE lncRNA exons (red), ancestral repeats (green) and four-fold degenerate sites (light blue) in individuals of Yoruba origin**. Click here for file
