# Supplementary material for: Mutations within lncRNAs are effectively selected against in fruitfly but not in human
Source: Genome Biol. 2013 May 27;14(5):R49. doi: 10.1186/gb-2013-14-5-r49 (PMC4053968; doi:10.1186/gb-2013-14-5-r49)
Supplement: Additional File 1 — Average phastCons scores across protein-coding (blue) and lncRNA (red) gene models in D. melanogaster (A) and human (B, C). Two hundred evenly-spaced nucleotides were randomly sampled per feature. The gray lines represent the 95% confidence intervals computed over 1,000 resampling. Average phastCons score for lncRNAs in human was computed over 200 randomly selected equidistant nucleotides within each of the categories. Confidence intervals were computed using 1,000 resampling of the data. [file gb-2013-14-5-r49-S1.PDF]

Figure: Average phastCons scores across protein-coding (blue) and lncRNAs (red) gene models in *D. melanogaster* (A) and human (B, C). Two hundred evenly spaced nucleotides were randomly sampled per feature. The grey lines represent the 95% confidence intervals computed over 1000 resampling.

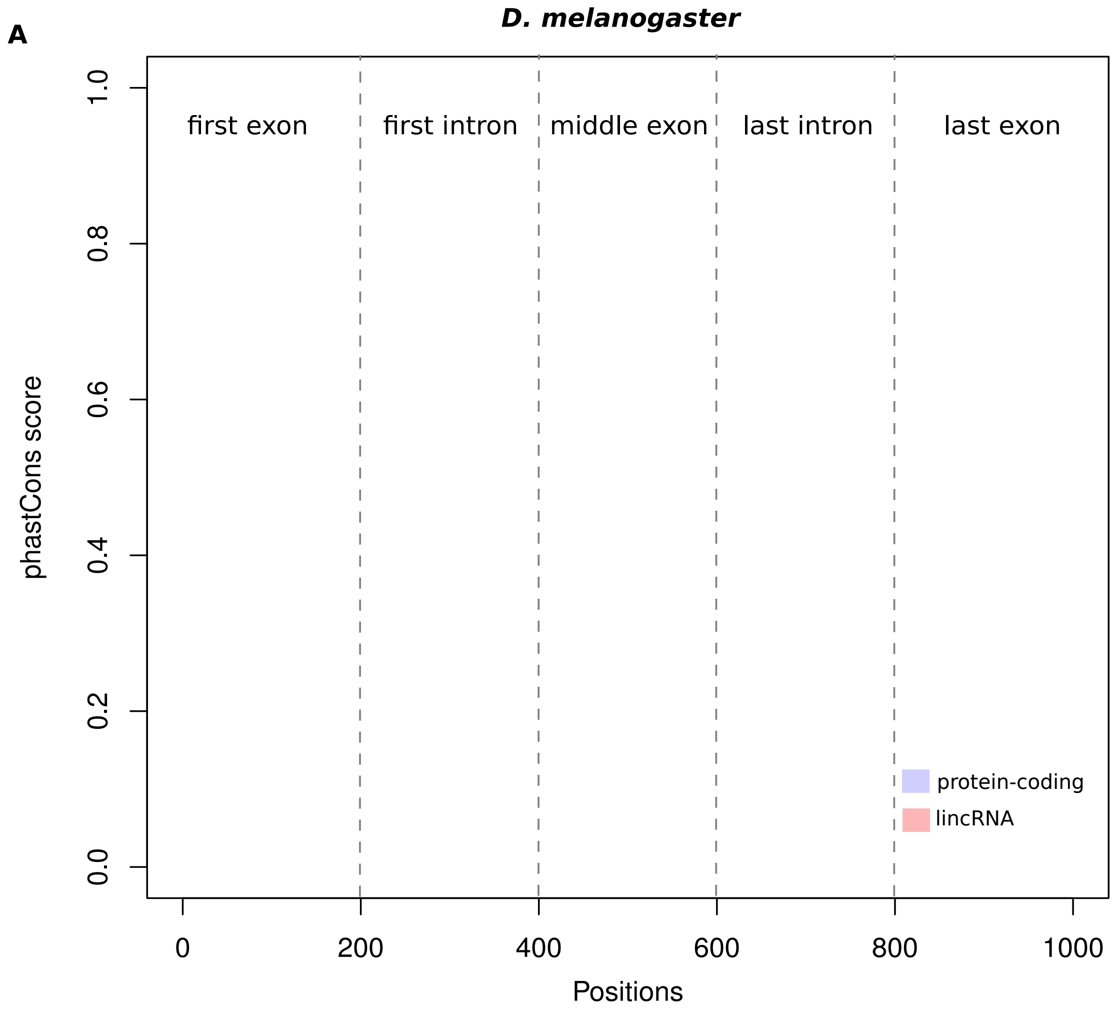

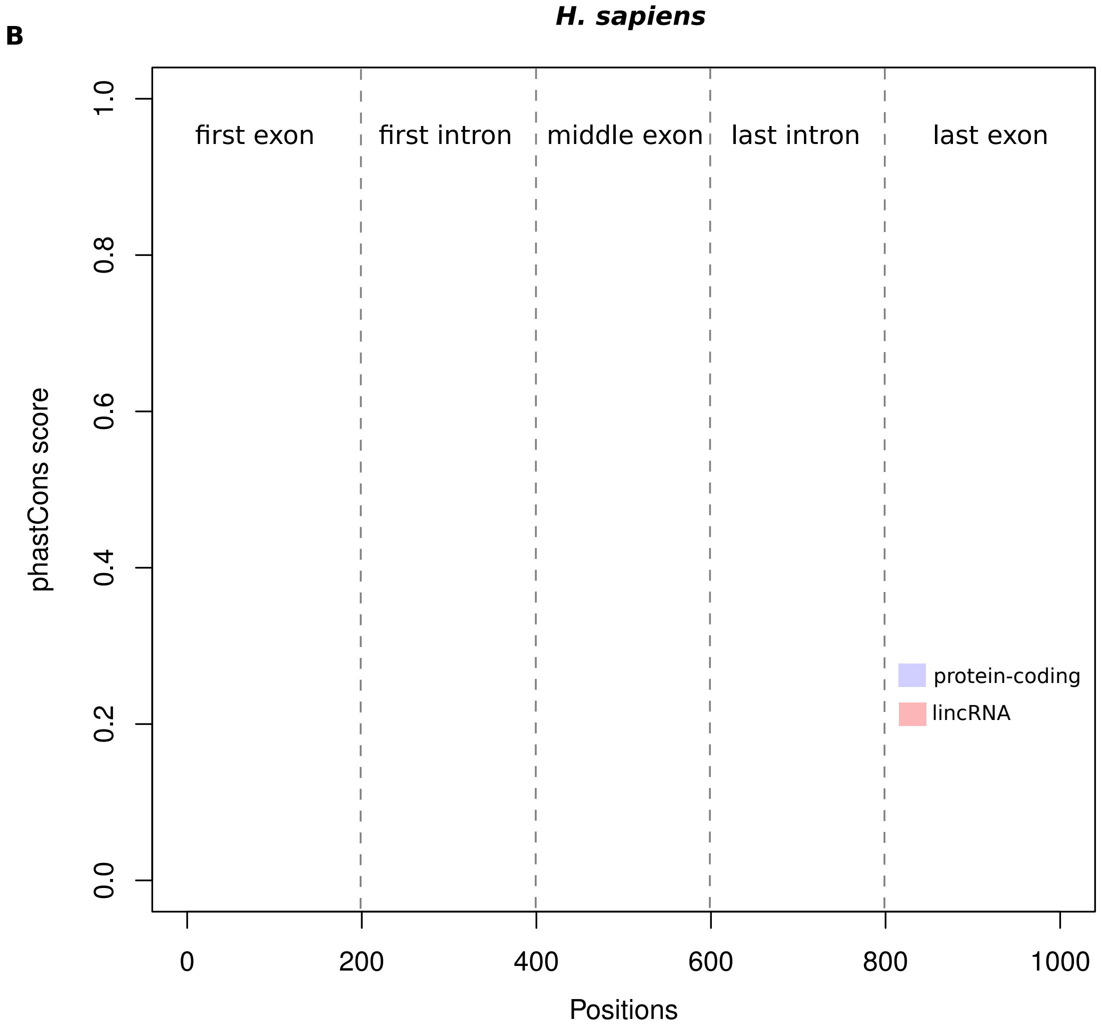

c

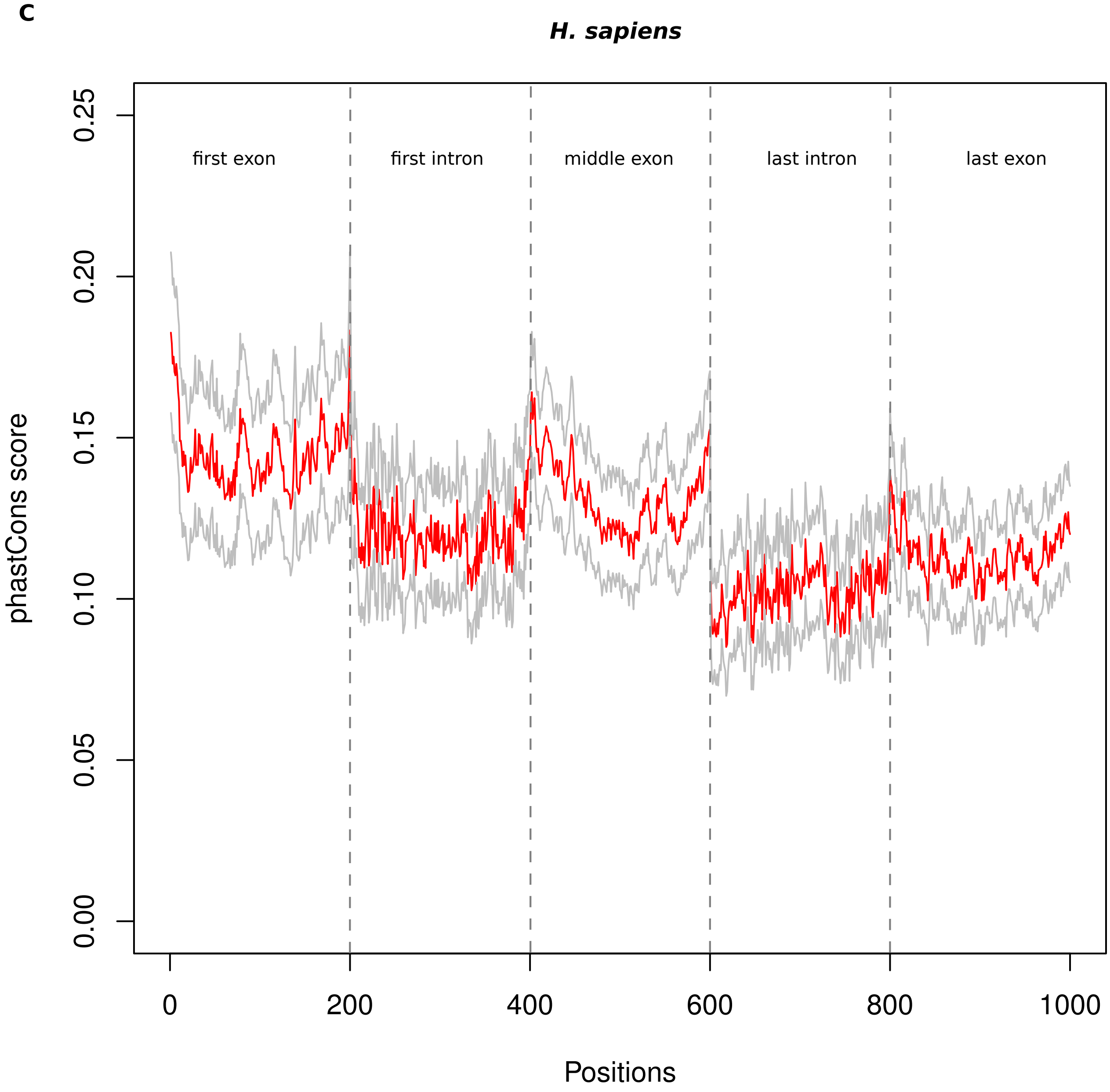
H. sapiens
first exon
first intron
middle exon
last intron
last exon
0.25
0.20
0.15
0.10
0.05
0.00
phastCons score
0
200
400
600
800
1000
Positions
