# Supplementary material for: Mutations within lncRNAs are effectively selected against in fruitfly but not in human
Source: Genome Biol. 2013 May 27;14(5):R49. doi: 10.1186/gb-2013-14-5-r49 (PMC4053968; doi:10.1186/gb-2013-14-5-r49)

Additional File 2: Median sequence conservation (phastCons) score across protein coding (blue) and positionally equivalent (PE) lncRNA (red) in human.

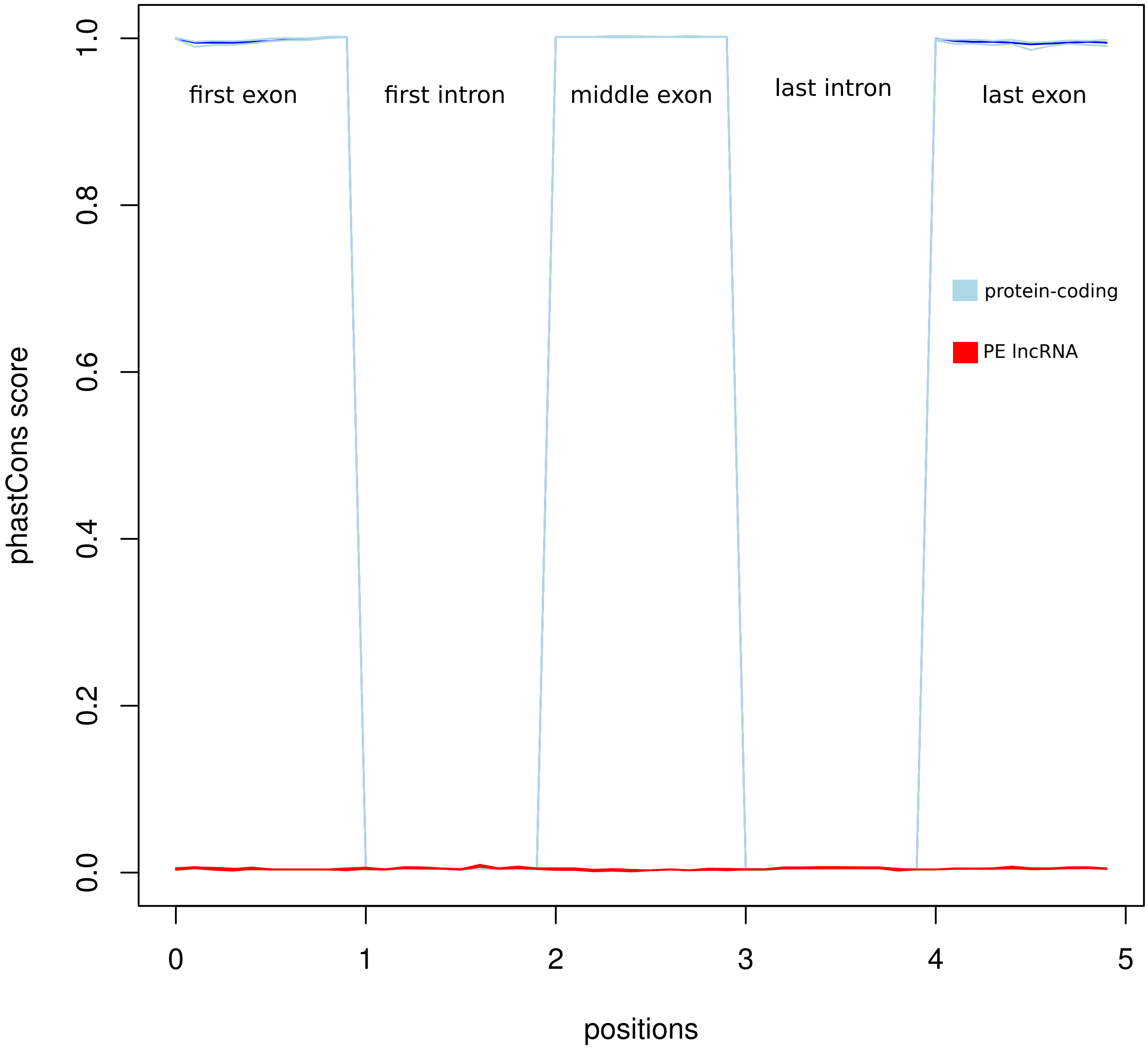

Supplement: Additional File 2 — Median sequence conservation (phastCons) score across protein coding (blue) and positionally equivalent (PE) lncRNA (red) in human. [file gb-2013-14-5-r49-S2.PDF]
