# Supplementary material for: Mutations within lncRNAs are effectively selected against in fruitfly but not in human
Source: Genome Biol. 2013 May 27;14(5):R49. doi: 10.1186/gb-2013-14-5-r49 (PMC4053968; doi:10.1186/gb-2013-14-5-r49)

Additional File 3: Comparison of protein-coding (blue) and lncRNA (red) 5' (A) and 3' (B) splice site conservation in *D. melanogaster*. Only protein coding sequences flanking lncRNAs were used in the analysis. The control set is based on the random selection of "GT" and "AG" dinucleotides within the intergenic sequence flanking the lncRNAs in *D. melanogaster*. The Simpson-Weaver index was computed for each sites using the alignments of each splice site and its neighbouring sequences with *D. simulans*, *D. sechellia D. yakuba* and *D. erecta* with Muscle.

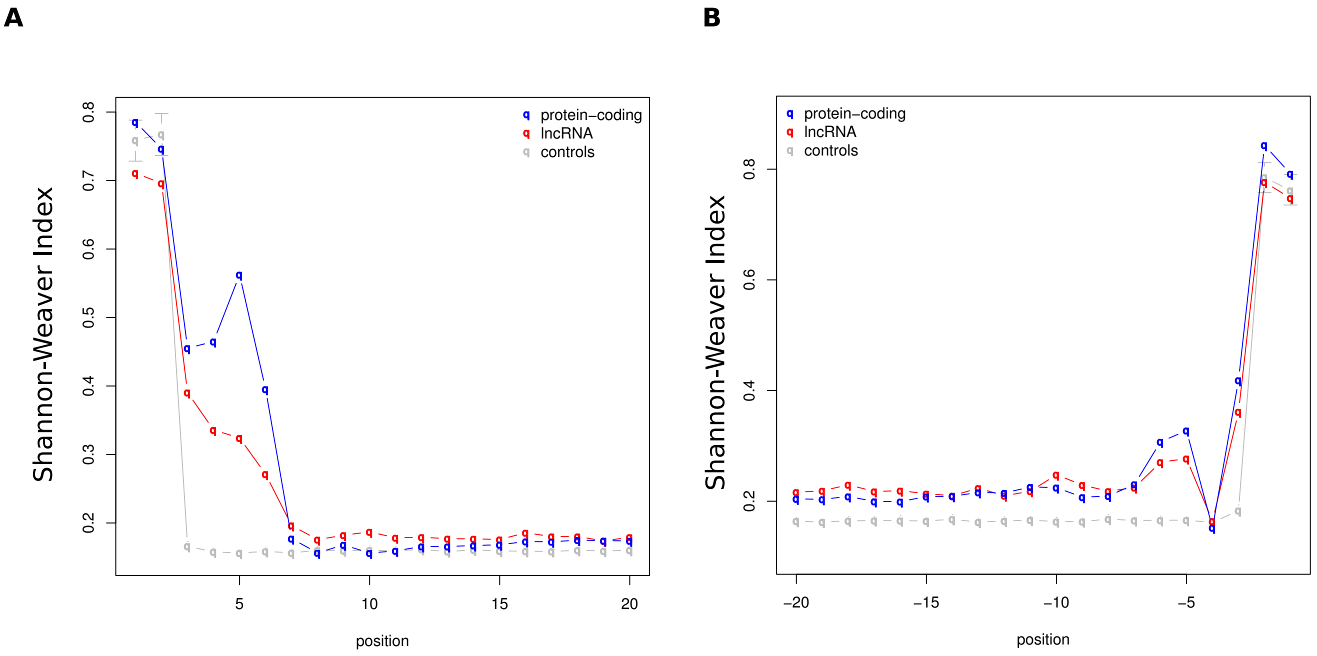

Supplement: Additional File 3 — Comparison of protein-coding (blue) and lncRNA (red) 5' (A) and 3' (B) splice site conservation in D. melanogaster . Only protein coding sequences flanking lncRNAs were used in the analysis. The control set is based on the random selection of 'GT' and 'AG' dinucleotides within the intergenic sequence flanking the lncRNAs in D. melanogaster. The Shannon-Weaver index was computed for each site using the alignments of each splice site and its neighbouring sequences with D. simulans, D. sechellia, D. yakuba and D. erecta with Muscle [102]. [file gb-2013-14-5-r49-S3.PDF]
