# Supplementary material for: Mutations within lncRNAs are effectively selected against in fruitfly but not in human
Source: Genome Biol. 2013 May 27;14(5):R49. doi: 10.1186/gb-2013-14-5-r49 (PMC4053968; doi:10.1186/gb-2013-14-5-r49)

Additional File 4: Distribution of the distances between consecutive SNPs within protein coding (black) and lncRNA (red) exons in *D. melanogaster*.

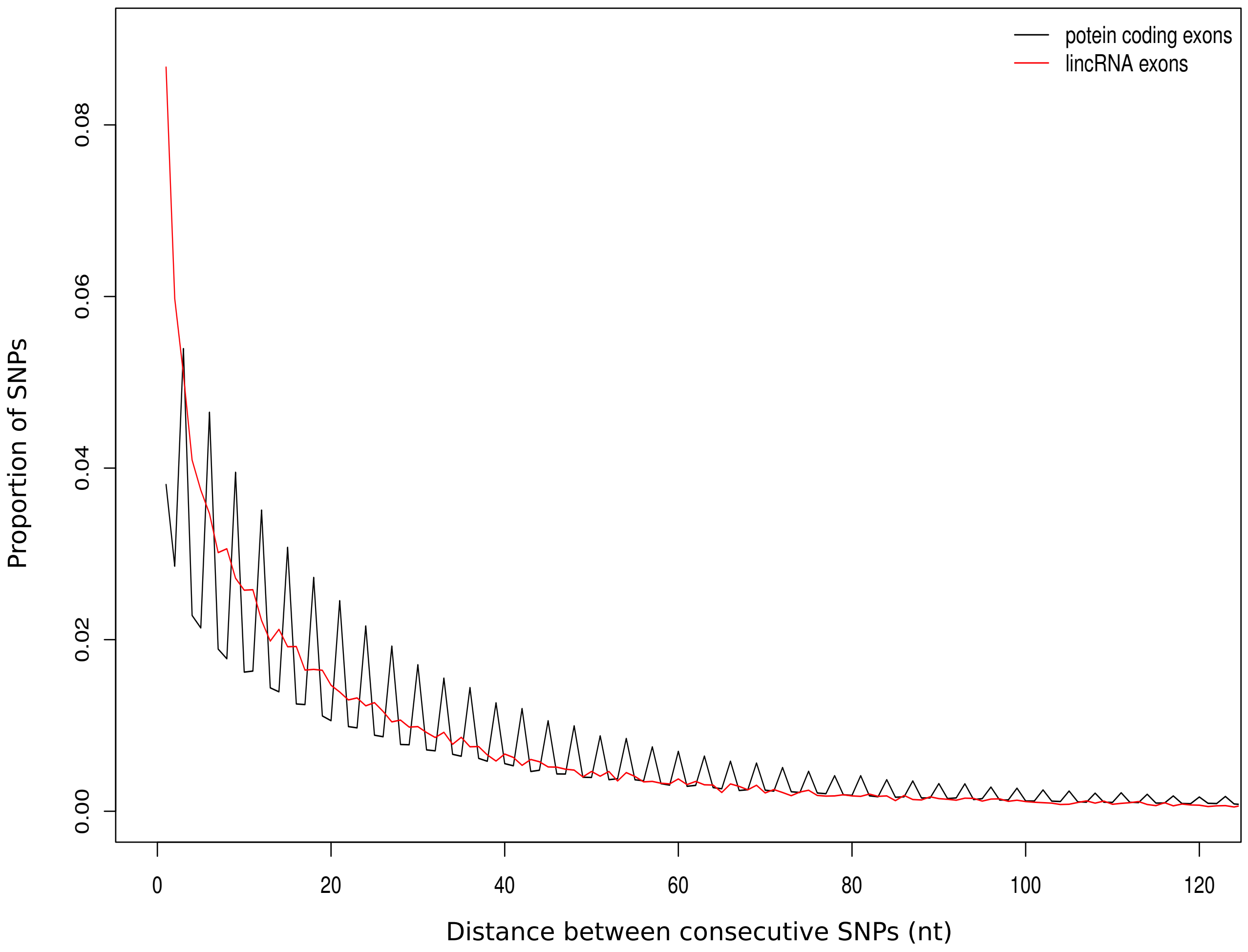

Supplement: Additional File 4 — Distribution of the distances between consecutive SNPs within protein coding (black) and lncRNA (red) exons in D. melanogaster. [file gb-2013-14-5-r49-S4.PDF]
