# Supplementary material for: Mutations within lncRNAs are effectively selected against in fruitfly but not in human
Source: Genome Biol. 2013 May 27;14(5):R49. doi: 10.1186/gb-2013-14-5-r49 (PMC4053968; doi:10.1186/gb-2013-14-5-r49)
Supplement: Additional File 5 — Average (standard deviation) polymorphism estimates for lncRNA and their flanking protein coding genes in human. PE: positional equivalent. A maximum distance threshold between lncRNA loci and ancestral sequences of 5 kb was applied. [file gb-2013-14-5-r49-S5.PDF]

Additional File 5. Average (standard deviation) polymorphism estimates for lncRNA and their flanking protein coding genes in human using a distance threshold of 5,000 nt. PE: positional equivalent.

	π_T	θ_T	Tajima's D	k
coding exons	$5.10 \times 10^{-4}(5.23 \times 10^{-4})$	$6.82 \times 10^{-4}(4.86 \times 10^{-4})$	$-0.41(0.84)$	$8.27 \times^{-4} (9.51 \times 10^{-4})$
coding introns	$8.99 \times 10^{-4}(4.84 \times 10^{-4})$	$9.97 \times 10^{-4}(4.28 \times 10^{-4})$	$-0.34(0.64)$	$1.32 \times^{-3} (8.46 \times 10^{-4})$
upstream coding	$9.89 \times 10^{-4}(9.88 \times 10^{-4})$	$9.91 \times 10^{-4}(6.93 \times 10^{-4})$	$-0.03(0.93)$	$1.34 \times^{-3} (1.52 \times 10^{-3})$
lncRNA exons	$1.05 \times 10^{-3}(8.41 \times 10^{-4})$	$1.16 \times 10^{-3}(6.60 \times 10^{-4})$	$-0.21(0.86)$	$1.58 \times^{-3} (1.52 \times 10^{-3})$
lncRNA introns	$1.03 \times 10^{-3}(7.91 \times 10^{-4})$	$1.09 \times 10^{-3}(5.49 \times 10^{-4})$	$-0.22(0.89)$	$1.48 \times^{-3} (1.21 \times 10^{-3})$
upstream lncRNA	$1.09 \times 10^{-3}(1.06 \times 10^{-3})$	$1.19 \times 10^{-3}(7.97 \times 10^{-4})$	$-0.14(0.92)$	$1.62 \times^{-3} (1.68 \times 10^{-3})$
PE lncRNA exons	$9.44 \times 10^{-4}(7.24 \times 10^{-4})$	$1.10 \times 10^{-3}(5.97 \times 10^{-4})$	$-0.26(0.90)$	$1.52 \times^{-3} (1.46 \times 10^{-3})$
PE lncRNA introns	$1.03 \times 10^{-3}(7.60 \times 10^{-4})$	$1.06 \times 10^{-3}(4.32 \times 10^{-4})$	$-0.17(0.79)$	$1.45 \times^{-3} (9.54 \times 10^{-4})$
Controls lncRNA exons	$1.02 \times 10^{-3}(8.13 \times 10^{-4})$	$1.16 \times 10^{-3}(6.55 \times 10^{-4})$	$-0.23(0.85)$	$1.46 \times^{-3} (1.51 \times 10^{-3})$
Controls lncRNA introns	$9.64 \times 10^{-4}(6.45 \times 10^{-4})$	$1.07 \times 10^{-3}(4.95 \times 10^{-4})$	$-0.28(0.75)$	$1.45 \times^{-3} (1.31 \times 10^{-3})$
ancestral repeats	$1.53 \times 10^{-3}(1.83 \times 10^{-3})$	$1.67 \times 10^{-3}(1.16 \times 10^{-3})$	$-0.12(0.92)$	$2.28 \times^{-3} (3.39 \times 10^{-3})$
