# Supplementary material for: Mutations within lncRNAs are effectively selected against in fruitfly but not in human
Source: Genome Biol. 2013 May 27;14(5):R49. doi: 10.1186/gb-2013-14-5-r49 (PMC4053968; doi:10.1186/gb-2013-14-5-r49)
Supplement: Additional File 6 — Average (standard deviation) polymorphism estimates for lncRNA and their flanking protein coding genes in human. PE: positional equivalent. A maximum distance threshold between lncRNA loci and ancestral sequences of 20 kb was applied. [file gb-2013-14-5-r49-S6.PDF]

Additional File 6. Average (standard deviation) polymorphism estimates for lncRNA and their flanking protein coding genes in human using a distance threshold of 20,000 nt. PE: positional equivalent.

	π_T	θ_T	Tajima's D	k
coding exons	$5.48 \times 10^{-4}(5.25 \times 10^{-4})$	$6.67 \times 10^{-4}(4.73 \times 10^{-4})$	$-0.38(0.84)$	$8.08 \times^{-4} (8.94 \times 10^{-4})$
coding introns	$9.03 \times 10^{-4}(4.71 \times 10^{-4})$	$1.0 \times 10^{-3}(4.12 \times 10^{-4})$	$-0.34(0.62)$	$1.32 \times^{-3} (8.11 \times 10^{-4})$
upstream coding	$1.03 \times 10^{-3}(9.99 \times 10^{-4})$	$1.04 \times 10^{-3}(7.57 \times 10^{-4})$	$-0.03(0.91)$	$1.49 \times^{-3} (1.75 \times 10^{-3})$
lncRNA exons	$1.07 \times 10^{-3}(8.99 \times 10^{-4})$	$1.17 \times 10^{-3}(6.96 \times 10^{-4})$	$-0.21(0.86)$	$1.59 \times^{-3} (1.57 \times 10^{-3})$
lncRNA introns	$1.03 \times 10^{-3}(7.73 \times 10^{-4})$	$1.11 \times 10^{-3}(5.59 \times 10^{-4})$	$-0.22(0.76)$	$1.47 \times^{-3} (1.2 \times 10^{-3})$
upstream lncRNA	$1.13 \times 10^{-3}(1.35 \times 10^{-3})$	$1.21 \times 10^{-3}(8.76 \times 10^{-4})$	$-0.13(0.93)$	$1.68 \times^{-3} (1.98 \times 10^{-3})$
PE lncRNA exons	$9.88 \times 10^{-4}(7.84 \times 10^{-4})$	$1.13 \times 10^{-3}(6.48 \times 10^{-4})$	$-0.25(0.90)$	$1.48 \times^{-3} (1.42 \times 10^{-3})$
PE lncRNA introns	$1.04 \times 10^{-3}(7.46 \times 10^{-4})$	$1.08 \times 10^{-3}(4.57 \times 10^{-4})$	$-0.19(0.77)$	$1.43 \times^{-3} (9.1 \times 10^{-4})$
Controls lncRNA exons	$1.05 \times 10^{-3}(8.63 \times 10^{-4})$	$1.16 \times 10^{-3}(6.65 \times 10^{-4})$	$-0.21(0.85)$	$1.45 \times^{-3} (1.51 \times 10^{-3})$
Controls lncRNA introns	$1.00 \times 10^{-3}(6.65 \times 10^{-4})$	$1.08 \times 10^{-3}(5.05 \times 10^{-4})$	$-0.23(0.75)$	$1.45 \times^{-3} (1.30 \times 10^{-3})$
ancestral repeats	$1.52 \times 10^{-3}(1.82 \times 10^{-3})$	$1.69 \times 10^{-3}(1.14 \times 10^{-3})$	$-0.12(0.92)$	$2.36 \times^{-3} (3.49 \times 10^{-3})$
