# Supplementary material for: Mutations within lncRNAs are effectively selected against in fruitfly but not in human
Source: Genome Biol. 2013 May 27;14(5):R49. doi: 10.1186/gb-2013-14-5-r49 (PMC4053968; doi:10.1186/gb-2013-14-5-r49)
Supplement: Additional File 7 — Comparison of derived allele frequency distribution of SNPs at non-synonymous sites (dark blue), within 3′ UTR (yellow), lncRNA exons (red), 5′ UTR, at four-fold degenerate sites (light blue), and within small introns in D. melanogaster. [file gb-2013-14-5-r49-S7.PDF]

Additional File 7:.Comparison of derived allele frequency distribution of SNPs at non-synonymous, 4-fold degenerate sites within protein-coding genes with lethal mutant phenotypes in *D. melanogaster* or annotated as being involved in genetic diseases or syndromes in human relative to the remaining protein-coding genes, lncRNA and neutrally evolving sequences.

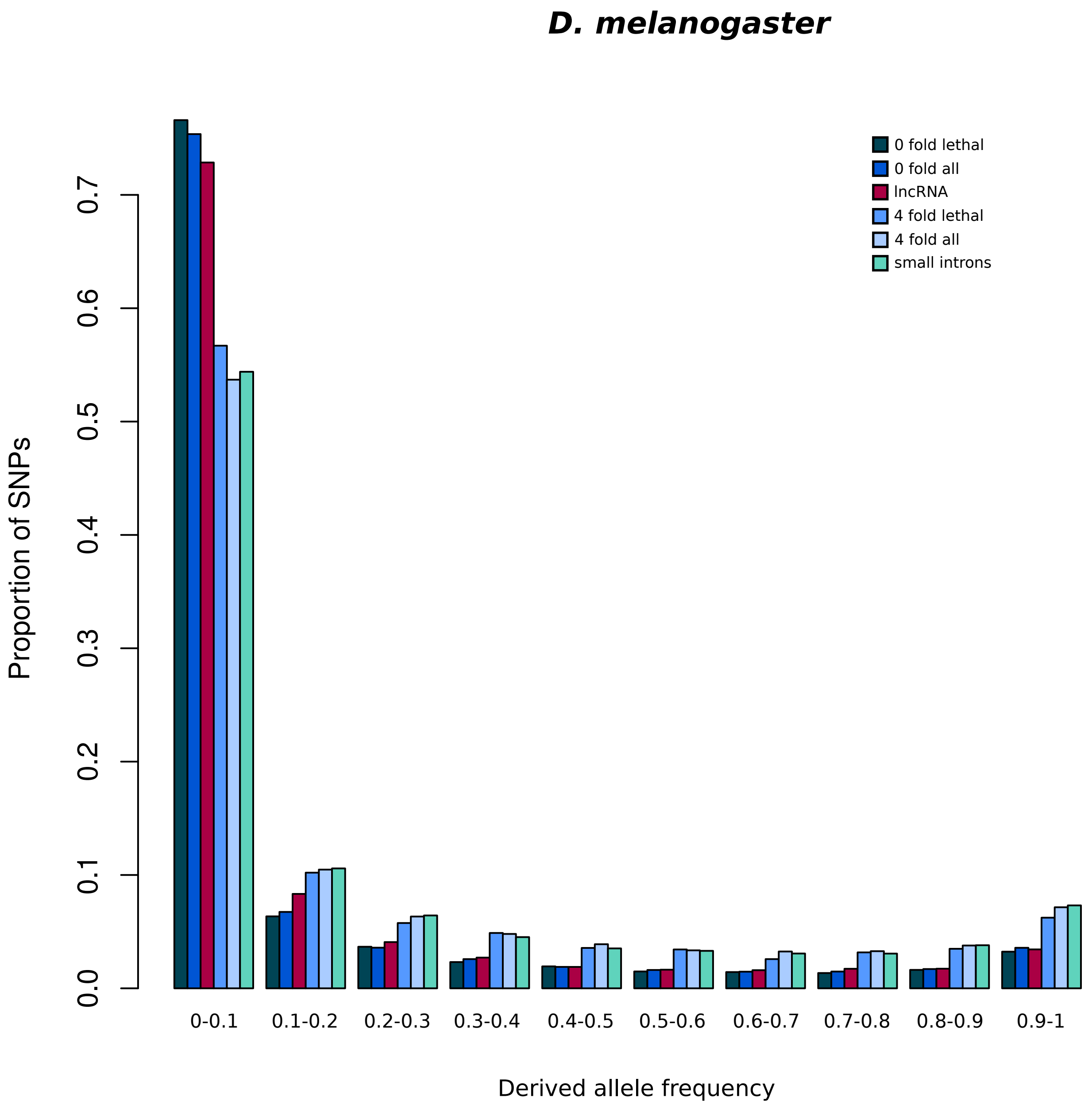

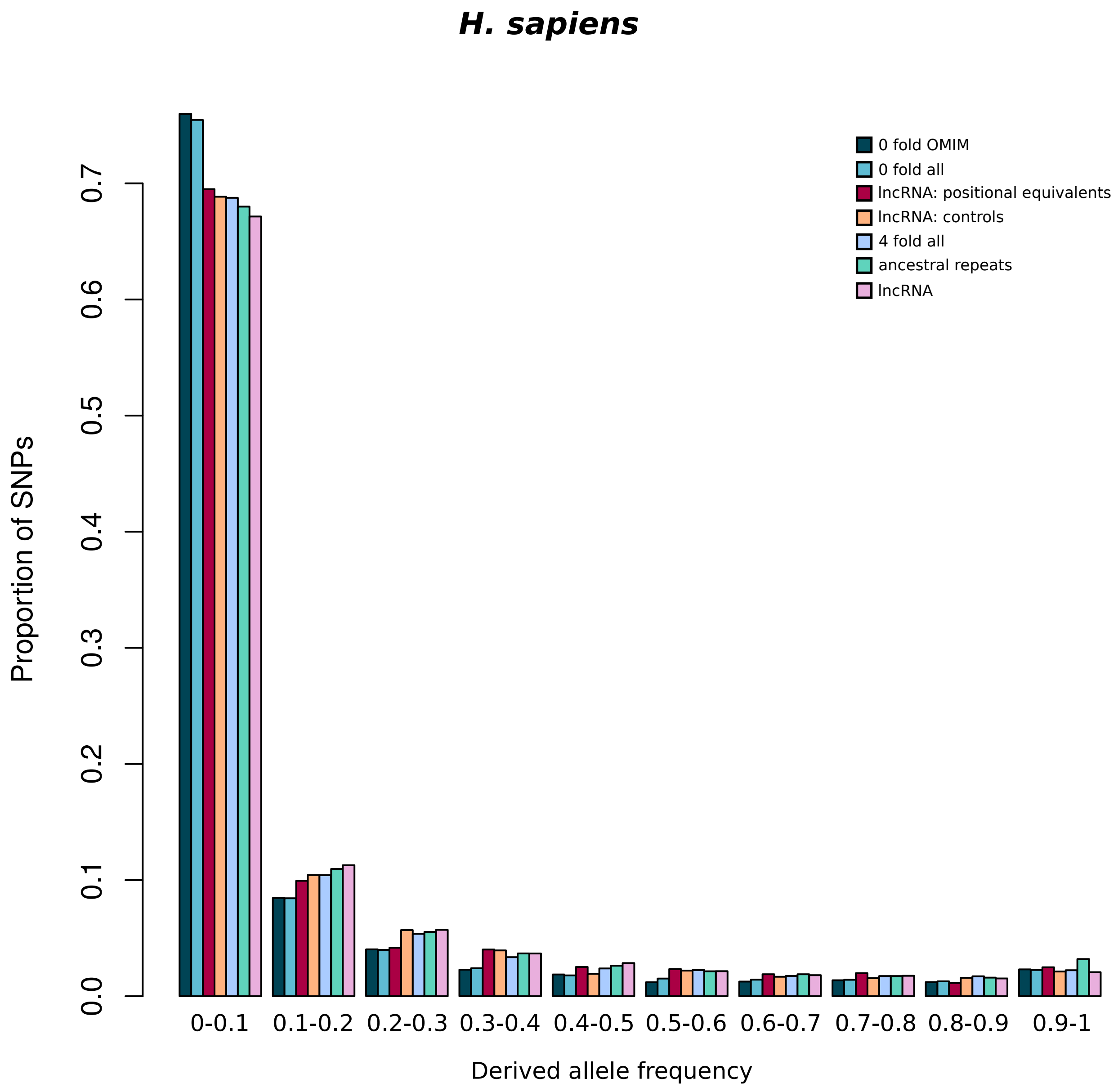

H. sapiens
0 fold OMIM
0 fold all
lncRNA: positional equivalents
lncRNA: controls
4 fold all
ancestral repeats
lncRNA
Proportion of SNPs
0.0
0.1
0.2
0.3
0.4
0.5
0.6
0.7
0-0.1
0.1-0.2
0.2-0.3
0.3-0.4
0.4-0.5
0.5-0.6
0.6-0.7
0.7-0.8
0.8-0.9
0.9-1
Derived allele frequency
