# Supplementary material for: Mutations within lncRNAs are effectively selected against in fruitfly but not in human
Source: Genome Biol. 2013 May 27;14(5):R49. doi: 10.1186/gb-2013-14-5-r49 (PMC4053968; doi:10.1186/gb-2013-14-5-r49)

Additional File 8: Derived allele frequency spectra for 0 fold, 4 fold degenerate sites, sites within lncRNA, sites upstream (400 nt) lncRNAs and protein coding genes in *D. melanogaster* (A) and human (B).

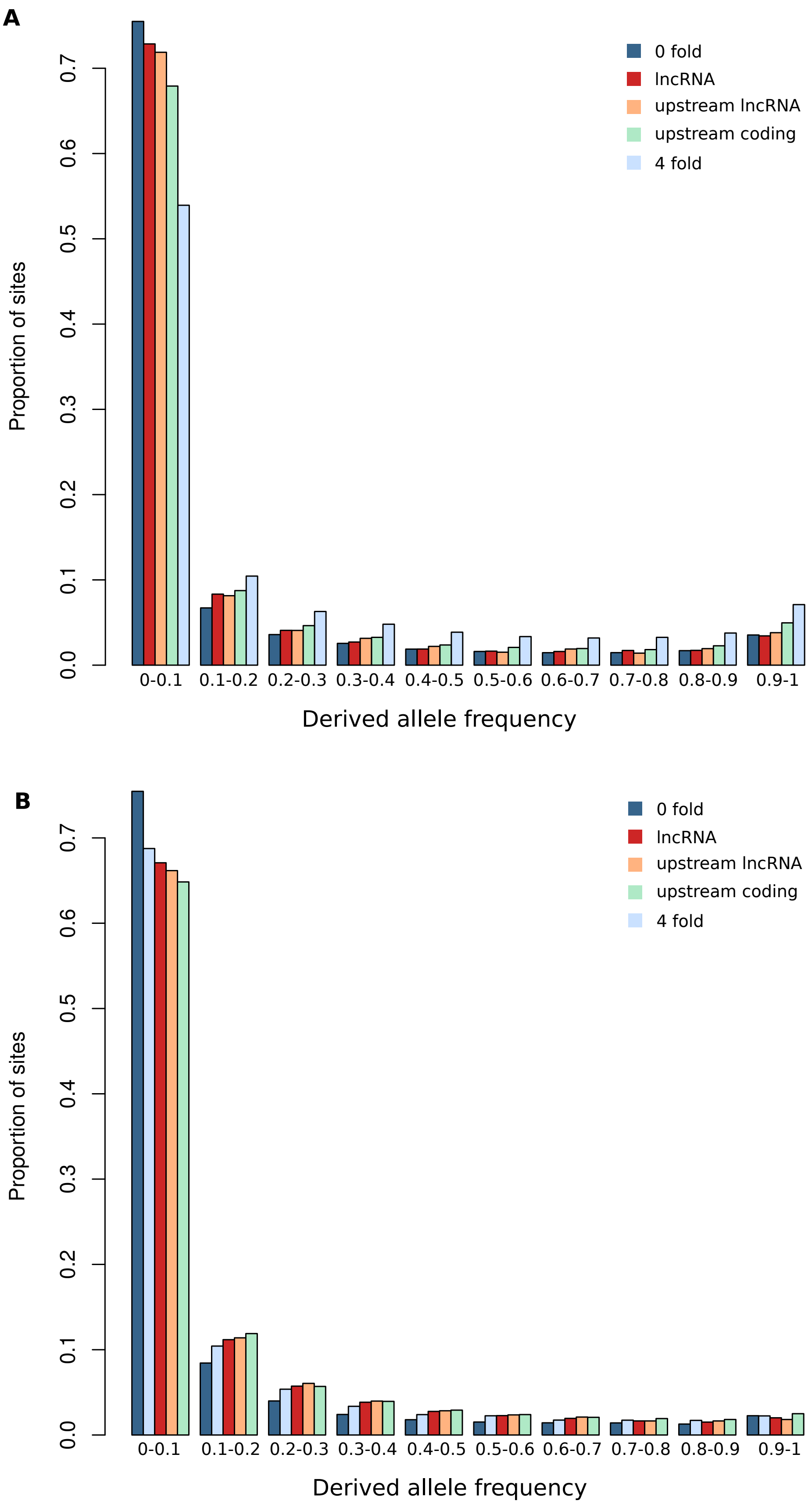

Supplement: Additional File 8 — Derived allele frequency spectra for 0-fold, four-fold degenerate sites, sites within lncRNA, sites upstream (400 nt) lncRNAs and protein coding genes in D. melanogaster (A) and human (B). [file gb-2013-14-5-r49-S8.PDF]
