# Supplementary material for: Mutations within lncRNAs are effectively selected against in fruitfly but not in human
Source: Genome Biol. 2013 May 27;14(5):R49. doi: 10.1186/gb-2013-14-5-r49 (PMC4053968; doi:10.1186/gb-2013-14-5-r49)

**Additional File 9.** Distribution of average conservation scores for intergenic lncRNAs in human

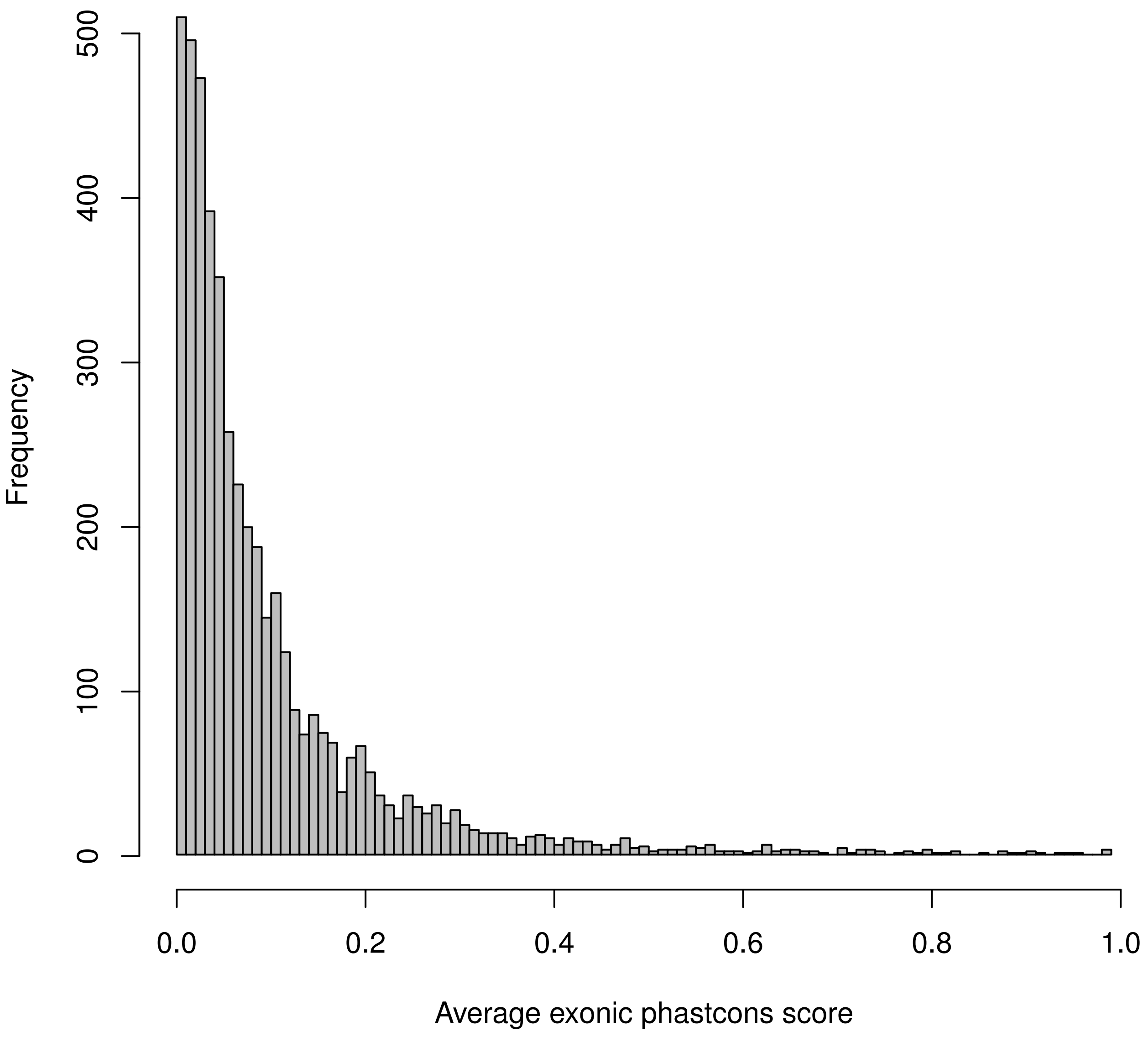

Supplement: Additional File 9 — Distribution of average conservation scores for intergenic lncRNAs in human. [file gb-2013-14-5-r49-S9.PDF]
