# Supplementary material for: Mutations within lncRNAs are effectively selected against in fruitfly but not in human
Source: Genome Biol. 2013 May 27;14(5):R49. doi: 10.1186/gb-2013-14-5-r49 (PMC4053968; doi:10.1186/gb-2013-14-5-r49)

Additional File 11. Comparison of derived allele frequency distribution of SNPs at 0-fold degenerate sites (blue), GENCODE lncRNA exons (red), ancestral repeats (green) and 4-fold degenerate sites (light blue) in individuals of Yoruba origin.

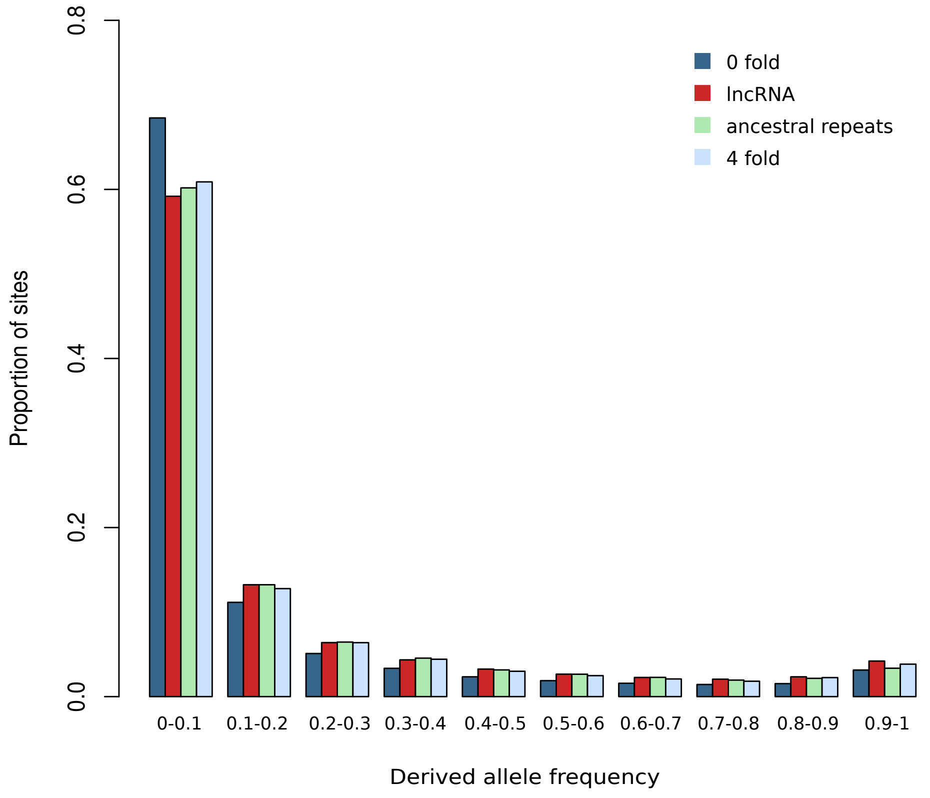

Supplement: Additional File 11 — Comparison of derived allele frequency distribution of SNPs at 0-fold degenerate sites (blue), GENCODE lncRNA exons (red), ancestral repeats (green) and four-fold degenerate sites (light blue) in individuals of Yoruba origin. [file gb-2013-14-5-r49-S11.PDF]
